# Hereditary methemoglobinemia diagnosed in adulthood: Type 1 cytochrome B5 reductase deficiency

**DOI:** 10.1007/s11845-026-04500-1

**Published:** 2026-06-19

**Authors:** Emine Afşin, Muzaffer Şenol, Ayşe Gürel, Emine Durak

**Affiliations:** 1 Department of Chest Diseases, Abant Izzet Baysal University, Bolu, Golkoy, 14200 Turkey; 2 Department of Chest Diseases, Gerede State Hospital, Bolu, Turkey; 3 Department of Medical Genetics, AIBU Izzet Baysal Training and Research Hospital, Bolu, Turkey; 4 Department of Hematology, AIBU Izzet Baysal Training and Research Hospital, Bolu, Turkey

**Keywords:** Hereditary methemoglobinemia, Type 1 cytochrome B5 reductase deficiency, Adulthood, Cyanosis

## Abstract

**Introduction:**

Type 1 CYB5R3 deficiency is a rare cause of hereditary methemoglobinemia. Although it is typically diagnosed during childhood, it may also be identified in older adults.

**Case:**

A 62-year-old male patient presenting with cyanosis and shortness of breath was diagnosed with Type 1 CYB5R3 deficiency. The diagnosis was supported by the chocolate-brown appearance of arterial blood, a methemoglobin (metHb) level of 17%, hypoxemia inconsistent with pulse oximetry findings, and the identification of a homozygous pathogenic mutation in CYB5R3 on sequence analysis. The patient is currently being followed with supplemental oxygen therapy and vitamin C treatment.

**Conclusion:**

In patients presenting with cyanosis and hypoxemia, arterial blood gas analysis should be evaluated for methemoglobinemia, as pulse oximetry may provide misleadingly high oxygen saturation readings. Congenital causes must be considered even in elderly patients.

## Introduction

Methemoglobin is formed when ferrous iron (Fe²⁺) in hemoglobin is oxidized to the ferric state (Fe³⁺). Because methemoglobin cannot bind oxygen, it is unable to deliver oxygen to tissues. Under normal conditions, blood methemoglobin (MetHb) levels remain below 1%, and elevations above this threshold lead to clinical manifestations. Although the acquired form of methemoglobinemia is more common, hereditary forms associated with biallelic variants in the nicotinamide adenine dinucleotide hydrogen (NADH)–dependent cytochrome B5 reductase 3 (CYB5R3) gene, or autosomal dominant variants in the globin genes, may also occur [1].

Type 1 CYB5R3 deficiency, characterized by isolated enzyme deficiency in erythrocytes, typically presents with mild symptoms such as cyanosis, fatigue, or exertional dyspnea, and patients are often asymptomatic for many years. In contrast, Type 2 deficiency, in which the enzyme deficiency affects multiple tissues including erythrocytes, liver, fibroblasts, and the brain, manifests with a severe clinical course accompanied by significant neurological impairment [2].We present this case because hereditary Type 1 methemoglobinemia was diagnosed during the evaluation of cyanosis in an adult patient.

## Case

**Fig. 1:**
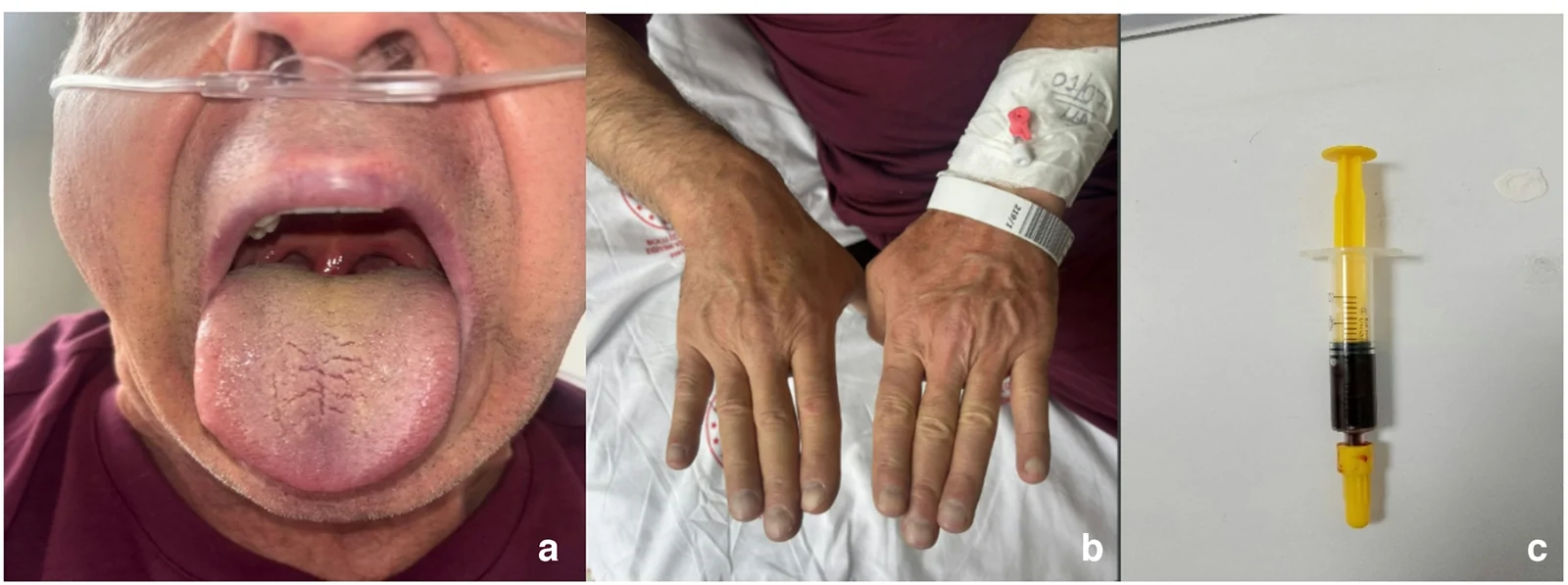
**a** Cyanosis of the lips, inside of the mouth, and tongue; **b**. Cyanosis and clubbing of the fingers; **c**. Arterial blood gas is chocolate brown

A 62-year-old man presented with a 3-day history of dyspnea that worsened with exertion. He was an active smoker with a 15 pack-year history. He had worked in a leather factory for 40 years and had left the job 8 months earlier. His medical history included hypertension, cerebrovascular disease, diabetes mellitus, and benign prostatic hyperplasia. His medications were acetylsalicylic acid, detrusitol, and doxazosin. He also had a history of repeated phlebotomies due to polycythemia. On physical examination, central and peripheral cyanosis were noted, along with digital clubbing (Fig. 1a, b). Systemic and respiratory examinations were otherwise unremarkable. Chest X-ray findings were normal. Oxygen saturation measured by pulse oximetry on room air was 89%. The patient reported that his lips and hands had been bluish-purple since childhood.

Arterial blood gas analysis revealed a pO₂ of 70.6 mmHg, an SO₂ of 77.7%, a pCO₂ of 35.4 mmHg, and a methemoglobin (MetHb) level of 17.4%. The arterial blood sample appeared chocolate brown in color (Fig. 1c). Hemogram results and liver and renal function tests were within normal limits. Pulmonary function testing was normal. Echocardiography showed mild mitral regurgitation, an ejection fraction of 60%, and a systolic pulmonary artery pressure of 31 mmHg.

**Fig. 2:**
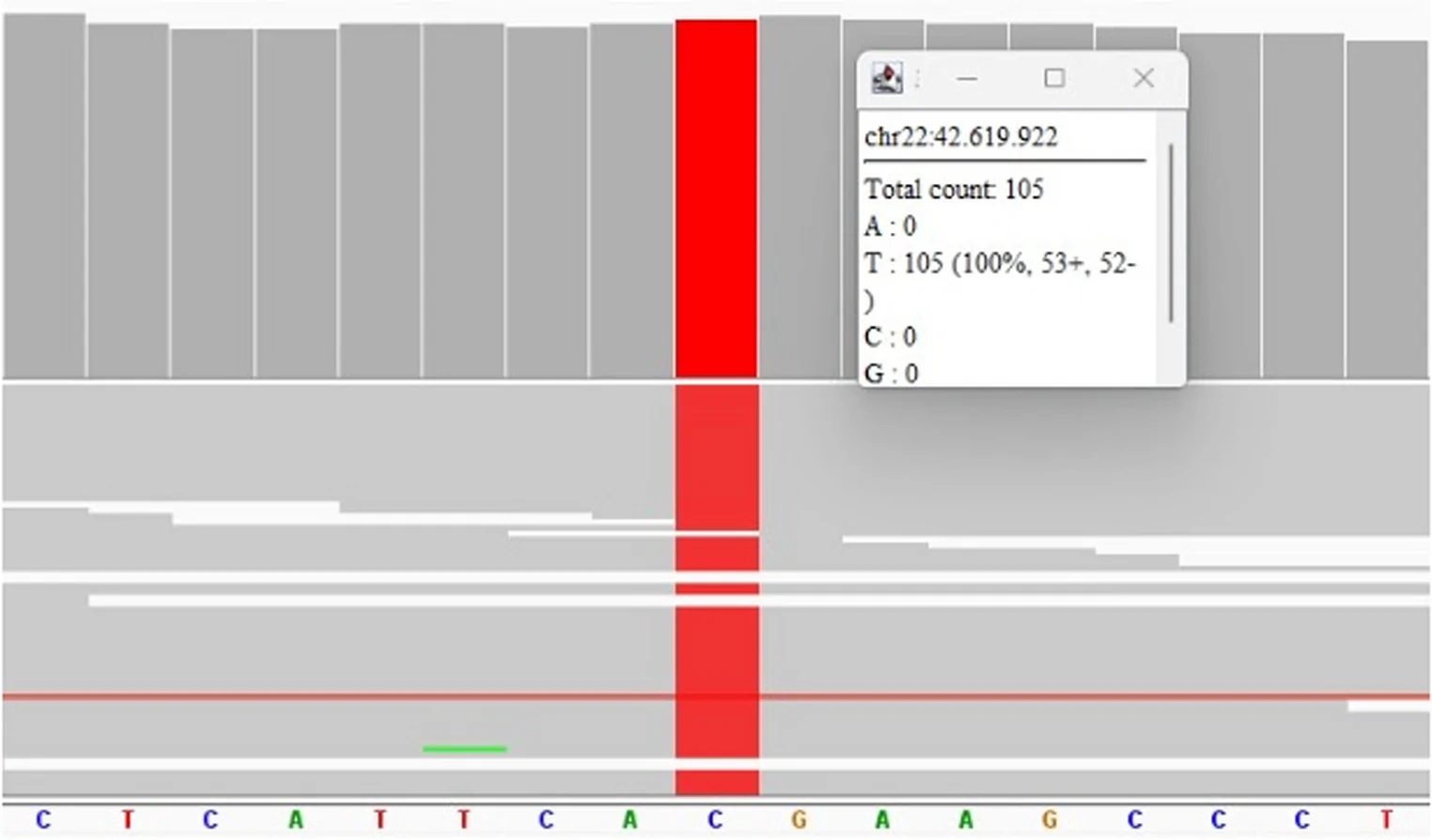
Integrated Genomics Viewer (IGV) image of the c.757G > A (p.Val253Met) variant detected by NGS analysis in the CYB5R3 (NM_000398.7) gene. The homozygous mutation located in exon 9 is shown in red. The CYB5R3 reference sequence corresponding to the region where the variant is located is provided below. Due to the presence of the reverse strand, the reference base “C” appears as the variant base “T” in the IGV image

Direct and indirect Coombs tests were negative. Glucose-6-phosphate dehydrogenase and haptoglobin levels were normal. Hemoglobin electrophoresis did not reveal any abnormal hemoglobin variants. The patient reported parental consanguinity. Genetic testing demonstrated a homozygous c.757G > A (p.Val253Met) pathogenic mutation in the CYB5R3 gene (NM_000398.7) (Fig. 2). Long-term oxygen therapy and vitamin C treatment were initiated.

## Discussion

In acquired methemoglobinemia, cyanosis typically appears shortly after exposure, so a detailed history of drug or toxin exposure must be investigated. A history of long-standing cyanosis, dark skin pigmentation in family members, or blue sclera should raise suspicion for congenital forms [3]. Acquired causes include local anesthetics (benzocaine, prilocaine, tetracaine, lidocaine), nitrates (nitroglycerin, inhaled nitric oxide, sodium nitroprusside), antibiotics (dapsone, rifampicin, sulfonamides, antimalarials), other medications (rasburicase, cyclophosphamide, metoclopramide), and environmental exposures such as fertilizers, herbicides, plastics, paints, and rubber products. Because our patient had worked in a leather factory for many years, occupational exposures were carefully evaluated. Leather processing environments are known to involve exposure to formic acid, hydrogen sulfide, sulfuric acid, chromium compounds, ammonia, toluene, benzene, and leather dust [4, 5]. Agents such as chlorobenzene, dinitrotoluene, and trinitrotoluene have been implicated in acquired methemoglobinemia. However, given that the patient had cyanosis since childhood and had been away from work for an extended period, occupational factors were excluded as the etiology. The medications used by the patient were also not associated with methemoglobinemia. Because the patient’s parents had a consanguineous marriage, congenital causes were considered primarily, and genetic analysis was performed.

Genetic testing confirmed a homozygous c.757G > A (p.Val253Met) mutation in the CYB5R3 gene through next-generation sequencing (NGS). This missense variant has previously been reported as pathogenic nine times in the ClinVar database and has been described in cases of both Type 1 and Type 2 methemoglobinemia [6]. In Type 1 CYB5R3 deficiency, cyanosis is common; however, due to compensatory erythrocytosis and chronic elevation of methemoglobin, tissue hypoxia occurs less frequently and symptoms tend to be milder [7]. Therefore, phlebotomy is not recommended in methemoglobinemia accompanied by polycythemia [3]. In our patient, polycythemia was present, and phlebotomies had been performed prior to diagnosis. Therefore, distinguishing whether polycythemia is primary or secondary is important, and phlebotomy should not be performed in every case of polycythemia.

Oxygen saturation measured by a pulse oximeter is determined by transmitting light at two wavelengths (red and infrared) through tissue—typically a finger or earlobe—and detecting the amount of light that is not absorbed by the tissue bed. Because this method is calibrated using data from healthy individuals with low carboxyhemoglobin and methemoglobin levels, pulse oximeters are unreliable in patients whose hemoglobin has altered absorption spectra. Methemoglobin has nearly identical absorption coefficients at both wavelengths, producing a 1:1 absorption ratio that corresponds to a saturation reading of approximately 85%. Therefore, when the true SO₂ is below 85%, methemoglobin may falsely elevate pulse oximetry readings [8]. In methemoglobinemia, despite the presence of cyanosis, arterial oxygen pressure remains within normal limits, although oxygen saturation appears low. In our patient, mild hypoxemia was present, and the saturation measured by pulse oximetry was higher than the true level.

The main morbidity associated with methemoglobinemia results from tissue hypoxia. Thus, asymptomatic patients may be monitored without additional treatment, and supplemental oxygen may be used when needed. Treatment is recommended for symptomatic patients or those with elevated MetHb levels. High methemoglobin levels are generally defined as > 10–30%, with values > 20% being the most commonly cited threshold in the literature [9]. In hereditary methemoglobinemia, higher MetHb levels are often better tolerated, and patients may remain asymptomatic even at levels between 30 and 40% [3]. There is no curative treatment for hereditary methemoglobinemia; oxidizing drugs should be avoided. Infants and pregnant women, in particular, should avoid well water or foods from areas where groundwater contamination with excessive nitrates (due to fertilizers or improper agricultural practices) is common.

For cosmetic concerns (blue discoloration), treatment options include ascorbic acid (300–500 mg/day, with or without methylene blue [MB]), methylene blue (100–300 mg/day), and riboflavin (60–120 mg/day) [2]. If repeated doses of MB do not result in improvement, therapeutic whole-blood exchange or hyperbaric oxygen therapy should be considered [10]. Since our patient had a relatively low MetHb level of 17% and cyanosis causing cosmetic concerns, vitamin C therapy was initiated. In addition, long-term oxygen therapy was started because of hypoxemia.

## Conclusion

Although pulmonary and cardiac causes are usually considered first in patients with cyanosis and hypoxemia, methemoglobinemia should also be included in the differential diagnosis. In particular, the presence of dark-colored arterial blood, pulse oximetry values that are inconsistent with arterial blood gas results, and a history of cyanosis since childhood should prompt evaluation for congenital causes. It was also emphasized that phlebotomy should not be performed in every case of polycythemia.

## Data Availability

The datasets used and/or analysed during the current study available from the corresponding author on reasonable request.

## References

[1] Prchal J (2021) Chapter 51: Methemoglobinemia and Other Dyshemoglobinemias, 10th edn. McGraw Hill, New York

[2] Ergün D, Narin E, Ergün R, Kanat F, Göksel B (2021) A rare cause of hypoxemia: hereditary methemoglobinemia. J Contemp Med 11(6):924–926

[3] Iolascon A, Bianchi P, Andolfo I, Russo R, Barcellini W, Fermo E et al (2021) Recommendations for diagnosis and treatment of methemoglobinemia. Am J Hematol 96(12):1666–1678. 10.1002/ajh.26340 PMC9291883 34467556

[4] Ministry of Labor and Social Security (2014)

[5] Ivek I, Knotek T, Ivičić T, Rubinić B, Bajlo P, Hamzić J (2022) Methemoglobınemıa - a case report and lıterature revıew. Acta Clin Croat 61(Suppl 1):93–98. 10.20471/acc.2022.61.s1.16 PMC9536159 36304805

[6] Kugler W, Pekrun A, Laspe P, Erdlenbruch B, Lakomek M. Molecular basis of recessive congenital methemoglobinemia, types I and II: Exon skipping and three novel missense mutations in the NADH-cytochrome b5 reductase (diaphorase 1) gene. Hum Mutat. 2001 Apr;17(4):348. 10.1002/humu.31 11295830

[7] Aldeeb M, Khalil IA, Yassin MA. Congenital Methemoglobinemia in a 33-Year-Old Patient: A Case Report on a Rare Presentation and a Review of the Literature. Cureus. 2023 Mar 10;15(3):e35974. 10.7759/cureus.35974 PMC10082972 37041911

[8] Barker SJ, Le N, Hyatt J (1991) Failure rates of transmission and reflectance pulse oximetry for various sensor sites. J Clin Monit 7:102–103

[9] Cortazzo JA, Lichtman AD (2014) Methemoglobinemia: a review and recommendations for management. J Cardiothorac Vasc Anesth 28(4):1043–7. 10.1053/j.jvca.2013.02.005 23953868

[10] Cho Y, Park SW, Han SK, Kim HB, Yeom SR (2017) A Case of Methemoglobinemia Successfully Treated with Hyperbaric Oxygenation Monotherapy. J Emerg Med 53(5):685–687. 10.1016/j.jemermed.2017.04.036 28838565

